# A randomised controlled trial of the clinical effectiveness, safety and cost-effectiveness of adalimumab in combination with methotrexate for the treatment of juvenile idiopathic arthritis associated uveitis (SYCAMORE Trial)

**DOI:** 10.1186/1745-6215-15-14

**Published:** 2014-01-09

**Authors:** Athimalaipet V Ramanan, Andrew D Dick, Diana Benton, Sandrine Compeyrot-Lacassagne, Dalia Dawoud, Ben Hardwick, Helen Hickey, Dyfrig Hughes, Ashley Jones, Patricia Woo, Clive Edelsten, Michael W Beresford

**Affiliations:** 1 University Hospitals Bristol NHS Foundation Trust, Upper Maudlin Street, Bristol BS2 8HW, UK; 2 Bristol Eye Hospital, Upper Maudlin Street, Bristol BS2 8HW, UK; 3 Great Ormond Street Hospital, Great Ormond Street, London WC1N 3JH, UK; 4 University of Liverpool, Alder Hey Children’s NHS Foundation Trust, Eaton Road, Liverpool L12 2AP, UK; 5 Bangor University, College Road, Bangor LL57 2DG, UK

**Keywords:** Adalimumab, Juvenile idiopathic arthritis, Methotrexate, Ophthalmology, Paediatric, Rheumatology, Safety, Uveitis

## Abstract

**Background:**

Juvenile idiopathic arthritis (JIA) is the most common rheumatic disease in children. Children with JIA are at risk of inflammation of the uvea in the eye (uveitis). Overall, 20% to 25% of paediatric uveitis is associated with JIA. Major risk factors for development of uveitis in JIA are oligoarticular pattern of arthritis, an age at onset of arthritis of less than seven years of age, and antinuclear antibody positivity. In the initial stages of mild to moderate inflammation the uveitis is asymptomatic. This has led to current practice of screening all children with JIA for uveitis. Approximately 12% to 38% of patients with JIA develop uveitis in seven years following onset of arthritis. In 30% to 50% of children with JIA-associated uveitis structural complications are present at diagnosis. Furthermore about 50% to 75% of those with severe uveitis will eventually develop visual impairment secondary to ocular complications such as cataract and glaucoma. Defining the severity of inflammation and structural complications in uveitis patients is now possible following Standardised Uveitis Nomenclature (SUN) guidelines, and modified to incorporate the consensus of end point and outcome criteria into the design of randomised trials. Despite current screening and therapeutic options (pre-biologics) 10% to 15% of children with JIA-associated uveitis may develop bilateral visual impairment and certified legally blind. To date, there remains no controlled trial evidence of benefits of biologic therapy.

**Methods/design:**

This study will randomise 154 patients aged 2 to 18 years with active JIA-associated uveitis (despite methotrexate (MTX) treatment for at least 12 weeks). All participants will be treated for 18 months, with follow up of 3 years from randomisation (continuing on MTX throughout). All participants will receive a stable dose of MTX and in addition either adalimumab (20 mg/0.8 ml for patients <30 kg or 40 mg/0.8 ml for patients weighing 30 kg or more, subcutaneous (s/c) injection every 2 weeks based on body weight), or placebo (0.8 ml as appropriate according to body weight) s/c injection every 2 weeks.

**Discussion:**

This is the first randomised controlled trial that will assess the clinical effectiveness, safety and cost effectiveness of adalimumab in combination with methotrexate for the treatment of juvenile idiopathic arthritis associated uveitis.

**Trial registration:**

ISRCTN10065623

## Background

Juvenile idiopathic arthritis (JIA) is the most common rheumatic disease in children. Children with JIA also are at risk of inflammation of the uvea in the eye (uveitis). Overall, 20% to 25% of all paediatric uveitis is associated with JIA [1,2], but a greater proportion is seen in referral cohorts. The major risk factors for the development of uveitis in JIA patients are oligoarticular pattern of arthritis, age at onset of arthritis younger than 7 years and antinuclear antibody positivity [3]. In the initial stages of mild to moderate inflammation, uveitis is entirely asymptomatic. This has led to the current practice of screening all children with JIA regularly for uveitis. Approximately 12% to 38% of patients with JIA will develop uveitis in the 7 years following the onset of arthritis [4,5]. In 30% to 50% of children with JIA-associated uveitis, structural complications are present at the time of diagnosis [6]. Furthermore, about 50% to 75% of those with severe uveitis will eventually develop visual impairment secondary to ocular complications such as cataract, glaucoma, band keratopathy and macular pathology [7-9].

Defining the severity of inflammation and structural complications in uveitis patients can now be more consistently described by following Standardised Uveitis Nomenclature (SUN) guidelines and incorporating them into the design of randomised controlled trials (RCTs) and cohort studies [10]. Significant poor prognosticators of poor visual acuity include structural changes at presentation, the need for intraocular surgery, posterior segment inflammation, abnormal intraocular pressure and failure to maintain long-term disease control as marked by persistent anterior chamber (AC) cell scores of 1 or higher [6-8,11]. Despite current screening and therapeutic options (prebiologics), 10% to 15% of children with JIA-associated uveitis may eventually develop bilateral visual impairment and become certified legally blind [12,13]. It is therefore critical to find more effective therapeutic interventions for them.

### Rationale

Methotrexate (MTX) is well-established as the first-line disease-modifyin\g agent in the management of JIA [14,15]. The current approaches to the treatment of mild JIA-associated uveitis include use of topical steroids. MTX is also thought to be effective for JIA-associated uveitis in children with moderate to severe uveitis [16-18], but there have been no prospective, randomised, placebo-controlled trials of MTX or steroid regimens in JIA-associated uveitis. Systematic review of the evidence for the effectiveness of MTX in JIA patients is restricted to joint involvement [14], but not in paediatric uveitis. Despite the scarce evidence, MTX has become the mainstay of treatment for JIA-associated uveitis [19]. However, about 15% to 50% of affected children will have refractory uveitis in spite of optimal therapy with MTX [16-18]. De Boer *et al*. [13] found that uveitis was not controlled in 30% of patients started on MTX during the first year of therapy and that, even when remission was achieved with MTX, 9 of 13 later relapsed and only 4 (18%) of 22 patients achieved total remission. In the Great Ormond Street cohort, a similarly low proportion of 12% were found to be in total remission 5 years after initiation of MTX therapy [20]. Several agents, including ciclosporin and mycophenolate mofetil (MMF), have been shown to be of benefit in controlling JIA-associated uveitis in small retrospective case series [21,22]. However, their use remains restricted because of intolerability due to adverse reactions and little evidence that they rescue MTX-refractory patients. In addition, neither ciclosporin nor MMF is very effective in controlling joint manifestations in children [19]. More recently, animal models and corroborative human evidence [23] support the role of tumour necrosis factor α (TNF-α) in the aetiopathogenesis of uveitis and, moreover, the potential value of inhibiting TNF-α as a therapeutic intervention [24].

Studies utilising experimental models of autoimmune uveitis have demonstrated that TNF-α plays a pivotal role in the pathogenesis of intraocular inflammation [23], which has been borne out in the treatment of adult uveitis [24]. In mouse models of anterior uveitis, deleting the p55 receptor, as well as combined TNF receptor (TNFR) p55- and p75-knockout animals, resulted in reduced disease [25] more significantly than the effect of TNFR p55 fusion protein [26]. Furthermore, in an animal model of uveitis, infliximab reduced disease severity [27], albeit at doses of 20 mg/kg. Translating these data to humans, several case series have been published that have demonstrated the efficacy of anti-TNF-α therapies, including infliximab and adalimumab, in the treatment of severe refractory uveitis in adults and children [28-33]. In contrast, etanercept has been reported not to halt the onset of uveitis or to be more effective than placebo [34,35]. It also has been shown to be less effective than infliximab in treating JIA-associated uveitis [31,36,37]. A number of reports of new-onset uveitis associated with etanercept use in JIA have been published [38]. Investigators in an AE register–based study who examined these cases determined that whilst the frequency was greater for etanercept than for infliximab or adalimumab (*n* = 20; four and two cases, respectively), causality could not be established [39]. Etanercept is not considered to be effective in treating intraocular inflammation [31].

Adalimumab is a fully human monoclonal antibody engineered by gene technology that uses site-directed mutagenesis to enhance its binding efficiency to TNF-α. It does not contain nonhuman or artificial protein sequences. Adalimumab binds only to TNF-α and has a half-life of approximately 2 weeks. The antibody has been studied extensively *in vitro* and *in vivo* and has been shown not to be toxic in animal toxicology experiments. A clinical trial of adalimumab as monotherapy or in combination with MTX in adult patients with rheumatoid arthritis showed a significant clinical response [40]. A multicentre, randomised, double-blind, stratified parallel group trial showed a significant benefit in children with active JIA [41].

Retrospective case series in paediatric noninfectious uveitis treated with adalimumab have shown very promising results, with 21 of 26 eyes from among 14 children with JIA-associated or idiopathic uveitis showing improvement in inflammation [42]. In another retrospective case series of 18 paediatric patients with uveitis, 88% had a substantial decrease in ocular inflammation, and adalimumab showed corticosteroid-sparing potential [28].

To the best of our knowledge, no prospective studies of the efficacy and safety of anti-TNF agents in JIA-associated uveitis have been conducted to date. In the RCT of adalimumab in JIA that demonstrated safety and efficacy, the most commonly reported AEs were infections and injection site reactions [41]. SAEs considered possibly related to the study drug by the investigators occurred in 14 patients. Seven of these AEs included one case of bronchopneumonia, herpes simplex infection, pharyngitis and pneumonia, and there were two cases of herpes zoster infection. In that trial, there were no deaths, malignant conditions, opportunistic infections, cases of tuberculosis (TB), demyelinating diseases or lupus-like reactions [41]. The fixed-dose model of 20 mg for children weighing <30 kg and 40 mg for children weighing ≥30 kg selected for our current SYCAMORE Trial is based on the data generated in the above Lovell *et al*. trial using the same dosing regimen [41].

### Potential risks and benefits

JIA-associated uveitis is a severe, potentially sight-threatening condition that is often inadequately treated using standard therapies. The advent of biologic therapies offers significant anticipated benefits. However, due care must be taken in determining the potential benefits of anti-TNF therapy, which is now being used off-label for this condition, against the potential associated risks. The safety (short- and long-term) of the new biologic therapies in children and young people is of major importance, particularly in our present study. The risk-to-benefit assessment of this intervention needs careful attention. Safety is therefore a key secondary outcome measure of the trial.

### Potential risks

The long-term follow-up of children on etanercept and adalimumab therapy described in the controlled studies published to date have not shown any increased risk of malignancies. However, the US Food and Drug Administration (FDA) recently issued an alert to healthcare professionals that its analysis has revealed that 48 children developed malignancies whilst on anti-TNF agents, and 11 of the children died [43]. The data are derived mainly from children and adolescents on etanercept and infliximab therapy; data on adalimumab are scarce because of limited follow-up. The analysis includes, in particular, children with Crohn’s disease. Of the 48 children, 88% were taking concomitant immunosuppressive medication, including azathioprine and MTX. The complete details of the FDA analysis are not currently available. Importantly, these data do not provide comparative information on long-term malignancy rates in JIA patients treated with MTX alone or in patients with untreated JIA. Subsequently, a presentation at the American College of Rheumatology (ACR)/Associate Rheumatology Health Professional 2009 Annual Scientific Meeting reported that in 1,168 patients over 16,396 patient-years, no increased risk of anti-TNF therapy in JIA patients was found [44]. Recent data presented at the European League Against Rheumatism emphasises the importance of comparing anti-TNF safety data to untreated disease [45] and that current data do not indicate a significant relative increase with respect to controls [46]. All these reports, however, emphasise the critical importance of making safety a major priority in this trial. This priority is both within the treatment and follow-up duration of the trial, but procedures are also in place to continue this safety follow-up for the longer term.

The risk of increased malignancy with azathioprine in patients with Crohn’s disease on infliximab is well-recognised [47,48]. As noted already, AEs associated with the recent adalimumab trial in JIA were associated with minimal safety signals [41]. A recent retrospective cohort study evaluated overall mortality and cancer mortality in relation to immunosuppressive drug exposure, including anti-TNF drugs, in adult patients with ocular inflammatory diseases [49]. The study did show an increased overall and cancer mortality in adult patients exposed to anti-TNF agents. The study’s authors acknowledge that these data need to be interpreted with caution because of the methodological issues associated with retrospective studies and the prevalence of comorbidity in patients taking anti-TNF drugs.

On the basis of adult patient data, as well as on the growing evidence base of published data derived from long-term follow-up in biologic registries, clinical trials and cohort studies, a number of important safety signals need to be considered in this trial. Patients taking TNF blockers are more susceptible to serious infections. Patients must therefore be monitored closely for infections, including TB, before, during and after treatment with adalimumab. Because the elimination of adalimumab may take up to 5 months, monitoring should be continued throughout this period.

AEs of the haematologic system, including medically significant cytopaenia (for example, thrombocytopaenia, leucopaenia) have been reported with adalimumab. All patients should be advised to seek immediate medical attention if they develop signs and symptoms suggestive of blood dyscrasias (for example, persistent fever, bruising, bleeding, pallor) while on adalimumab.

Adalimumab monotherapy, as well as concomitantly with MTX, has been studied in rheumatoid arthritis, polyarticular JIA and psoriatic arthritis patients [41]. Antibody formation to adalimumab itself was lower when adalimumab was given together with MTX compared to adalimumab monotherapy. Administration of adalimumab without MTX resulted in increased formation of antibodies, increased clearance and reduced efficacy of adalimumab. In patients with polyarticular JIA, adalimumab antibodies were identified in 27 (15.8%) of 171 patients treated with adalimumab. In patients not given concomitant MTX, the incidence of adalimumab antibodies was 22 (25.6%) of 86, compared to 5 (5.9%) of 85 when adalimumab was used as an add-on to MTX.

Patients who develop a new infection while undergoing treatment with adalimumab should be monitored closely and should undergo a complete diagnostic evaluation. Administration of adalimumab should be discontinued if a patient develops a new serious infection or sepsis, and appropriate antimicrobial or antifungal therapy should be initiated until the infection is controlled. Physicians should exercise caution when considering the use of adalimumab in patients with a history of recurring infection or with underlying conditions which may predispose them to infections, including the use of concomitant immunosuppressive medications. Serious infections seen in clinical trials include pneumonia, pyelonephritis, septic arthritis and septicaemia.

### Known potential benefits

In rheumatoid arthritis phases I to IV studies [50], all individual components of the adult ACR response criteria (number of tender and swollen joints, physician and patient assessment of disease activity and pain, disability index Health Assessment Questionnaire scores and C-reactive protein (mg/dl) levels) improved at 24 or 26 weeks compared to placebo. In these studies, adalimumab-treated patients achieved statistically significant improvement in their rheumatoid arthritis symptoms based on ACR criteria showing 20% improvement (ACR20) and ACR50 responses compared to placebo as early as 1 to 2 weeks after initiation of treatment.

In polyarticular course JIA, adalimumab has been shown to have a significant clinical benefit in improving JIA on the basis of core paediatric ACR response criteria [51]. In the double-blind withdrawal design phase of the trial of adalimumab in JIA patients [41], amongst patients not receiving MTX, there was a significant increase in the number of disease flares in those patients who subsequently received placebo compared to those given adalimumab (71% vs. 43%; *P* = 0.03) [52]. In those patients receiving concomitant MTX, flares occurred in 65% on placebo compared to 37% who received adalimumab (*P* = 0.02). At 48 weeks, the percentage of patients treated with MTX who had ACR Pediatric 30 response criteria (Pedi 30), Pedi 50, Pedi 70 and Pedi 90 responses was significantly greater for those treated with adalimumab than for those given placebo (ACR Pedi 30: 63% vs. 38%, *P* = 0.03; ACR Pedi 50: 63% vs. 38%, *P* = 0.03; ACR Pedi 70: 63% vs. 27%, *P* = 0.002). Open-label extension of the studies showed sustained responses for up to 104 weeks of treatment. As outlined in the protocol rationale, its reported use in JIA-associated uveitis warrants a RCT trial to assess its clinical effectiveness and safety.

## Methods/design

### Trial design

**Figure 1:**
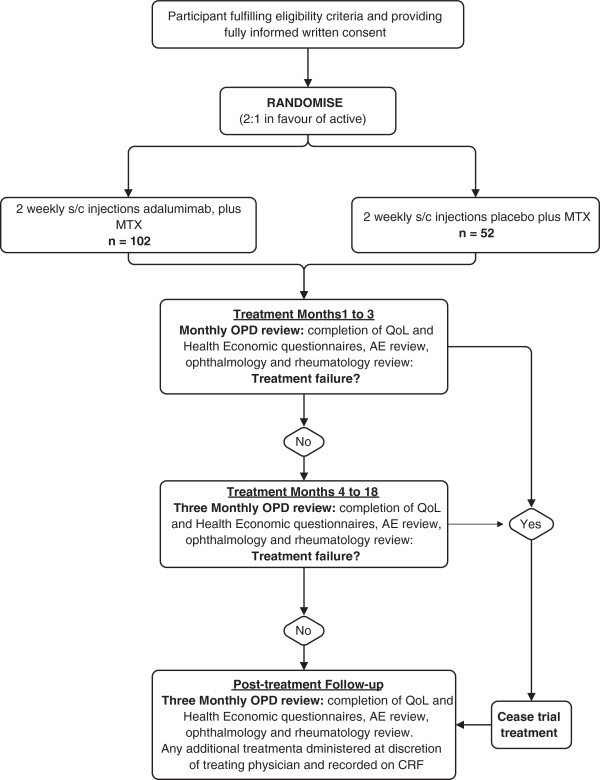
**Schematic of trial design and patient journey.** AE, adverse event; CRF, case report form; MTX, methotrexate; OPD, Outpatient Department; QoL, quality of life.

The SYCAMORE Trial will randomise 154 patients ages 2 to 18 years with active JIA-associated uveitis (despite MTX treatment for at least 12 weeks). All participants will be treated for 18 months, with follow-up of 3 years from randomisation (continuing on MTX throughout). All participants will receive a stable dose of MTX as well as either adalimumab (20 mg/0.8 ml for patients <30 kg or 40 mg/0.8 ml for patients weighing 30 kg or more, subcutaneous injection every 2 weeks based on body weight) or placebo (0.8 ml as appropriate according to body weight) by subcutaneous injection every 2 weeks. Figure 1 shows the schematic of trial design.

### Regulatory approval

Full ethical approval was granted by the London Hampstead Research Ethics Committee (11/LO/0425). Full approval also was given by the Medicines and Healthcare Products Regulatory Agency (EudraCT 2010-021141-41).

### Primary end point

The primary end point of the study is ‘time to treatment failure’. *Treatment failure* is defined by one or more of the following factors:

1. Anterior segment inflammatory score grade (SUN criteria)

a. Two-step increase from baseline in SUN cell activity score (AC cells) over two consecutive readings

b. Sustained nonimprovement with entry grade of 3 or greater for 2 consecutive readings

c. Only partial improvement (+1 grade) or no improvement from baseline with development of other ocular comorbidities (defined below) that are sustained

d. Worsening of existing (upon enrolment) ocular comorbidities (defined below) after 3 months

e. Sustained scores recorded at entry grade measured over two consecutive readings (grade 1 or 2) still present after 6 months of therapy

In addition, following at least 3 months of therapy, treatment failure is met if any of the following factors are met:

2. Use of concomitant medications: at any time, requirement for concomitant medications in a manner outside predefined acceptable criteria or for any of the concomitant medications not allowed

3. Intermittent or continuous suspension of study treatment (adalimumab or placebo) for a cumulative period longer than 4 weeks

Ocular comorbidities are defined as follows: (1) disc swelling and/or cystoid macular oedema as gauged clinically and, where possible, by optical coherence tomography (OCT); and/or (2) raised intraocular pressure (>25 mmHg) sustained over two consecutive visits without any response to a single ocular hypotensive agent; and/or (3) hypotony (<6 mmHg) sustained over two consecutive visits; and/or (4) development of unexplained reduction in vision of 15 letters according to the logarithm of the minimal angle resolution (logMAR) over two consecutive visits (if the patient has cataracts, they will remain in the trial, even if cataract surgery is required; failure will remain as described in the end points described above).

Where a reading is must be sustained over two consecutive visits to define treatment failure, the time of treatment failure will be taken as the second of these readings.

### Secondary end points

1. Number of participants failing treatment

2. Incremental cost-effectiveness and cost utility of adalimumab added to MTX compared with MTX alone

3. Health status according to the multiattribute Health Utility Index Mark 2

4. Safety, tolerability and compliance, defined as follows:

a. Adverse events (AEs) and serious adverse events (SAEs)

b. Laboratory parameters (haematological and biochemical analysis and urinalysis)

c. Development of human antihuman antibody to adalimumab determined with samples collected at 1, 6 and 18 months

d. Participant diaries and dosing records will determine tolerability and compliance throughout the trial treatment period

5. Use of corticosteroids over the duration of the study period and throughout follow-up, including the following:

a. Total oral corticosteroid dose

b. Reduction and reduction rate of systemic corticosteroid dose from entry dose

c. Topical corticosteroid use (frequency) compared to use at time of entry

d. Need for pulsed corticosteroid

6. Optic and ocular outcomes, defined as follows:

a. Number of participants with disease flares (defined by worsening based on SUN criteria) following a minimum of 3 months of disease control

b. Number of participants with disease flares within the first 3 months of the study

c. Visual acuity as measured by age-appropriate logMAR assessment

d. Number of participants with resolution of associated optic nerve or macular oedema (as assessed by slit-lamp biomicroscopy or OCT (where available)

e. Number of participants with disease control (defined as zero cells with topical treatment for 3 and 6 months)

f. Number of participants entering disease remission (defined as zero cells without topical treatment for 3 and 6 months)

g. Duration of sustaining inactive disease (zero cells with or without topical treatment)

7. Quality of life assessments (Childhood Health Questionnaire and Childhood Health Assessment Questionnaire)

8. ACR Pedi core set criteria at ACR30, ACR50, ACR70, ACR90 and ACR100 levels

9. Number of participants with disease flares, in remission on and off medication [53], related to their JIA and with minimum disease activity [54]

10. Number of participants requiring change in biologic and/or disease-modifying antirheumatic drug therapy for arthritis due to failure to respond

### Study population

#### Inclusion criteria

Participants are eligible for the trial based upon having at least one eye fulfilling the following eligibility criteria:

1. Children and young people ages 2 years and older up to and including 18 years of age and fulfilling the International League Against Rheumatism diagnostic criteria for JIA (all subgroups with uveitis)

2. At the time of trial screening, participants must have active anterior uveitis, defined as a sustained grade of cellular infiltrate in anterior chamber of SUN criteria grade ≥1+ or more during the preceding 12 weeks of therapy despite MTX and corticosteroid (both systemic and topical) therapy

3. They must have failed MTX therapy previously (minimum dose of 10 to 20 mg/m^2^ with a maximum dose of 25 mg/participant)

4. Participants must have been on MTX for at least 12 weeks and on a stable dose for 4 weeks prior to the screening visit; omission of a maximum of 2 weeks of MTX treatment within these 12 weeks is acceptable and will not render patients ineligible, unless the omission occurs within the 4 weeks prior to the screening visit

5. No disease-modifying immunosuppressive drugs, other than MTX, in the 4 weeks prior to screening

6. Written informed consent of participants or their parents or legal guardians, as well as assent where appropriate

7. Participants and their parents or legal guardians must be willing and able to comply with protocol requirements

8. For participants of reproductive potential (males and females), use of a reliable means of contraception throughout trial participation; postpubertal females must have a negative serum pregnancy test within ten days before receiving the first dose of the trial drug

9. Ability to be randomised and commence the trial treatment within 2 weeks of the screening visit

### Exclusion criteria

The following are the exclusion criteria:

1. Uveitis without a diagnosis of JIA

2. Currently on adalimumab therapy or previous adalimumab treatment

3. Treated with another biologic agent within the previous five half-lives of the agent

4. More than six topical steroid eye drops per day prior to screening (This dose must have been stable for at least 4 weeks prior to the screening visit.)

5. Patients on prednisone or a prednisone equivalent, change of dose within 30 days prior to screening

6. Patients on prednisone or a prednisone equivalent at a dose >0.2 mg/kg/day

7. Intraarticular joint injections within 4 weeks prior to screening

8. Any ongoing chronic or active infection (including infective uveitis), any major episode of infection requiring hospitalisation or any treatment with intravenous antibiotics within 30 days or oral antibiotics within 14 days prior to the screening evaluation

9. History of active TB requiring less than 6 months of treatment or history of untreated latent TB

10. History of central nervous system (CNS) neoplasm, active CNS infection, demyelinating disease or any progressive or degenerative neurological disease

11. Poorly controlled diabetes or persistent, poorly controlled, severe hypertension (>95th percentile for height and age) as determined by the treating physician

12. Previous history of malignancy

13. Intraocular surgery within the 3 months prior to screening (cataract, glaucoma or vitrectomy)

14. Intraocular or periocular corticosteroids within 30 days prior to screening

15. History of ocular herpetic disease

16. Pregnant or nursing female

17. Demonstration of clinically significant deviations in any of the following laboratory parameters:

a. Platelet count <100,000/mm^3^

b. Total white blood cell count <4,000 cells/mm^3^

c. Neutrophils <1,000 cells/mm^3^

d. Aspartate aminotransferase or alanine aminotransferase more than twice the upper limit of normal (ULN) or serum bilirubin more than twice the ULN

e. Glomerular filtration rate (GFR) <90 ml/min/1.73 m^2^ (GFR (ml/min/1.73 m^2^ bovine serum albumin) = 0.55 × height (cm)/plasma creatinine (mg/dl))

f. Hematocrit <24%

18. Live or attenuated vaccine received within 3 months prior to screening

19. Previous randomisation into either arm of the SYCAMORE Trial

20. Intraocular pressure <6 mmHg or intraocular pressure >25 mmHg

21. Intraocular pressure control requiring more than one topical pressure-lowering therapy or requiring systemic acetazolamide

### Selection of centres/clinicians

The study will be initiated at centres once all their global requirements (for example, local research and development (R&D) approval) and study-specific conditions (for example, training requirements) have been met and all necessary documents have been returned to the Medicines for Children Research Network Clinical Trials Unit (MCRN CTU). Initiation meetings will cover the requirements outlined in the Clinical Trials Research Centre’s standard operating procedures related to site training and setup.

### Centre/clinician inclusion criteria

The following are the inclusion criteria for centres and clinicians:

1. Centres offering a combined paediatric rheumatology/ophthalmology service

2. All participants recruited should have regular and emergency access to a paediatric rheumatologist and/or ophthalmologist

3. Completion of calibration training in ophthalmology assessments

4. Sufficient demonstrated capacity of staff to carry out study assessments

5. Curriculum vitae (CV), including a record of International Conference for Harmonisation (ICH) of Good Clinical Practice (GCP) training of the Principal Investigator (PI)

6. CV including a record of ICH GCP training of other personnel on the delegation log

7. Completion and return of the ‘Signature and Delegation Log’ to the CTU

8. Positive site-specific information

9. Local R&D approval

10. Signed contract between site and sponsor

11. Receipt of evidence of completion of criteria 8 to 10 by CTU

12. Ability to perform biochemical assessments

All sites are expected to demonstrate the ability to run paediatric clinical trials in accordance with GCP and as such demonstrate support and infrastructure for all aspects of trial delivery, including integration of the clinical research teams with pharmacy, clinical laboratory and research support services. All centres will be expected to work in collaboration with MCRN CTU support where present, including the National Institute for Health Research MCRN local research networks, the Comprehensive Local Research Network and their equivalents in Scotland, Wales and Northern Ireland.

### Centre and clinician exclusion criteria

Centres and clinicians will be excluded if they do not meet the inclusion criteria and expectations stated above.

### Sample size

The sample size is based on data regarding failure rates from 62 patients on MTX in a comparable population provided by Dr C Edelsten of Great Ormond Street Hospital. After 3 months, 11 patients had disease control based on grade 0 SUN criteria (18%), and therefore, on the basis of the trial inclusion criteria, they would not be eligible for inclusion in the trial. At 15 months following the start of treatment with MTX, 23 patients of the 51 who had failed at 3 months had achieved disease control (45%), leaving 28 (55%) who had not. The null hypothesis underlying this trial is that there is no significant difference between adalimumab and placebo in controlling disease activity of JIA-associated uveitis unresponsive to MTX therapy.

To detect a relative reduction of 50% between a failure rate of 60% and 30% with 90% power, at 5% significance and 2:1 randomisation, a total of 140 patients (93 adalimumab and 47 placebo) are required. There is unlikely to be a trial of this nature again in the near future; therefore, we have increased the power of the study to 90% from the conventional power level of 80% to optimise the detection of a significant difference between treatment regimens if one truly exists. A trial of adalimumab in JIA with or without MTX powered the study using a 40% absolute (57% relative) difference in the rate of flare between the placebo and adalimumab groups [41].

The advent of biological therapies in JIA has led international investigators to a paradigm shift in the treatment of JIA and its related complications, leading to significantly more ambitious outcomes in clinical trials, including elimination of inflammation and normalisation of short-term and long-term function [15,55]. To this end, instead of previously accepted clinical outcomes of 30% absolute difference in JIA patient outcomes between active agent and placebo [56], increasingly significant differences are being expected and regarded as significant, with new definitions of response being established for use in clinical trials, such as clinical remission and minimal disease activity [53,54]. Indeed, 40% of patients in the adalimumab JIA trial were reported as showing an ACR Pedi 100 response (100% response rate) at 2 years [41].

The clinically relevant outcomes of JIA-associated uveitis may take years to develop, and the relationship between isolated measures of clinical activity and long-term outcomes remains ill-defined. Recent studies suggest that the length of continuously controlled activity is likely to be of more clinical relevance than short-term improvements in activity levels.

In view of these factors, as well as the expectation expressed unanimously through consumer consultation in the development of this trial protocol, we have set a minimum 50% relative difference in failure rates between interventions. We estimate that loss to follow-up will be approximately 10% based on (1) the severe nature of the disease potentially resulting in loss of vision, (2) clinical opinion arising from an *a priori* meeting of investigators representing participating centres, (3) feedback from consumer representatives and (4) the exisiting experience of the investigators and consumer representatives with compliance with current use of biologic therapies in JIA-associated uveitis. Therefore, we increased the sample size by approximately 10%, giving us a total of 154 patients (102 adalimumab and 52 placebo).

### Randomisation

Randomisation will be undertaken during normal working hours (Monday to Friday from 0900 to 1700) by the pharmacy departments of participating centres upon receipt of a randomisation request form and prescriptions from authorised clinicians. Pharmacy personnel will verify that these documents are appropriately completed before proceeding. The PI and delegated research staff are responsible for (1) notifying pharmacy personnel of potential randomisations so that they can ensure adequate drug supplies are available on-site and (2) completing the appropriate trial documents and delivering these to the pharmacy department at their centres so that pharmacy personnel can proceed with randomisation.

Participants will be randomised using a secure (24-hour) web-based randomisation programme. Randomisation lists will be generated at a 2:1 ratio in favour of the active therapy. The lists will incorporate random elements, and the web randomisation programme will be controlled centrally by the MCRN CTU. Both measures are being employed to ensure that participant allocations are concealed. Participant treatment allocation will be displayed on a secure website, and an automated email confirmation will be sent to the authorised randomiser. In the event of an internet connection failure between the centre and the randomisation system, the centre should contact the MCRN CTU immediately to try to resolve the problem. If this is not possible within a reasonable amount of time, then the supplied backup randomisation envelopes will be used to provide the treatment allocation.

### Assessments and procedures

After written consent (and assent where appropriate) from the parent or legal guardian or from the trial participant, is obtained, medical and ophthalmic histories will be taken and recorded on the appropriate case report form (CRF) with particular emphasis on other disorders of relevance and allergies. Separate sections on the CRF will be provided to record the JIA and uveitis-specific medical and ophthalmic histories and the participant’s other medical and surgical histories. Medication use (prescriptions, over-the-counter medicines and herbal supplements) during the 4 weeks prior to the screening visit will also be recorded. Information from a physical examination, measures of disease activity and complications, laboratory tests (haematological and biochemical analysis and urinalysis) and medication and surgical histories will be gathered at the screening visit and again at each subsequent trial visit.

Protocol assessments will be performed according to the table of assessments (Table 1).

**Table 1:** **Study visits and assessments**^**a**^

		**Time (months)**															
		**Randomisation and commencement of treatment**								**End of treatment**						**End of trial**	
**Events and assessments**	**Screening**^**b**^	**0**^**c**^	**1**	**2**	**3**	**6**	**9**	**12**	**15**	**18**	**21**	**24**	**27**	**30**	**33**	**36**	**Premature withdrawal**
Written informed consent	X																
Confirmed consent (verbal)		X	X	X	X	X	X	X	X	X	X	X	X	X	X		
Assessment of eligibility criteria	X	X															
Review of medical/ophthalmic/surgical history	X																
Review of concomitant medications	X	X	X	X	X	X	X	X	X	X	X	X	X	X	X	X	X
Pregnancy test (serum)		(X)			X	X	X	X	X	X	X						
Purified protein derivative tuberculin skin test^d^/test for latent TB as locally performed	X																
Urinalysis^e^	X		X	X	X	X	X	X	X	X	X	X	X	X	X	X	X
Randomisation		X															
Study intervention		X	X	X	X	X	X	X	X	X							
Compliance with study intervention		X	X	X	X	X	X	X	X	X							
Physical examination: complete	X		X	X	X	X	X	X	X	X	X	X	X	X	X	X	X
Vital signs (heart and respiratory rates, temperature, blood pressure)	X		X	X	X	X	X	X	X	X	X	X	X	X	X	X	X
Height	X		X	X	X	X	X	X	X	X	X	X	X	X	X	X	X
Weight	X		X	X	X	X	X	X	X	X	X	X	X	X	X	X	X
Childhood Health Questionnaire		X	X	X	X	X	X	X	X	X							
Childhood Health Assessment Questionnaire		X	X	X	X	X	X	X	X	X							
Health Utilities Index Mark 2 questionnaire		X			X	X	X	X		X			X			X	X
Client Service Receipt Inventory		X															
Sample for DNA collection	(X)				(X)					(X)							
RNA and serum/plasma	(X)				(X)					(X)							
Haematological analysis	X		X	X	X	X	X	X	X	X	X	X	X	X	X	X	X
Biochemical analysis	X		X	X	X	X	X	X	X	X	X	X	X	X	X	X	X
Samples for HAHA analyses^f^	X				X												
ANA, dsDNA and ENA	X							X									
Ophthalmic assessments																	
Vision assessment	X		X	X	X	X	X	X	X	X	X	X	X	X	X	X	X
Optical coherence tomography (optional)	(X)		(X)	(X)	(X)	(X)	(X)	(X)	(X)	(X)	(X)	(X)	(X)	(X)	(X)	(X)	(X)
Assessment of vitritis	X		X	X	X	X	X	X	X	X	X	X	X	X	X	X	X
Slit-lamp biomicroscopy	X		X	X	X	X	X	X	X	X	X	X	X	X	X	X	X
Cataract scoring	X		X	X	X	X	X	X	X	X	X	X	X	X	X	X	X
Goldmann tonometry or Tono-Pen	X		X	X	X	X	X	X	X	X	X	X	X	X	X	X	X
Standard ACR paediatric core set outcome variables	X		X	X	X	X	X	X	X	X	X	X	X	X	X	X	X
Tanner score	X				X			X			X	X	X	X	X	X	X
Review of participant diaries	X		X	X	X	X	X	X	X	X	X	X	X	X	X	X	(X)
Assessment of adverse events	X		X	X	X	X	X	X	X	X	X	X	X	X	X	X	X

^a^ACR, American College of Rheumatology; ANA, antinuclear antibody; dsDNA, double-stranded DNA; ENA, extractable nuclear antigen; HAHA, human antiadalimumab antibody to adalimumab; TB, tuberculosis; (X), as applicable/indicated/appropriate. ^b^All procedures should be done before study intervention. ^c^Visit 0 must be completed and treatment must be commenced within 14 days of the screening visit (10 days for pregnancy test). ^d^Participants who are PPD-positive at screening will require a chest X-ray. Treatment of participants who have a positive PPD skin test and/or abnormal chest X-ray should be carried out in accordance with regional and/or national guidelines and initiated at least 4 weeks prior to the first dose of trial medication. Participants with recent (within 6 months of trial entry screen) positive PPD test (≥5 mm) who are being treated with appropriate prophylaxis may request a waiver for a PPD screening from the Medicines for Children Research Network Clinical Trials Unit. Documentation of the positive PPD test should be available, as well as chest X-ray reports, from the date of the positive PPD test and treatment or prophylaxis history from near the time of the participant’s conversion. ^e^Microscopic urinalysis will be obtained at baseline and for other visits only if relevant abnormalities greater than trace are noted on the dipstick analysis. ^f^To be done also as required if anaphylaxis occurs during trial.

With regard to treatment timelines, ‘1-month treatment’ is defined as 4 weeks. After treatment is commenced, the appointments for each subsequent visit should be made for 4 weeks or 3 months (12 weeks) afterwards, depending on the visit schedule. An allowance of −7 days or +7 days will be allowed for monthly visits and −15 days or +15 days for the 3-month visits. Should unscheduled visits be required for any reason, they will be recorded on the ‘unscheduled visit’ CRF. To define treatment failure, there should be an interval of at least 4 weeks between assessments.

### Analysis plan

The primary analysis will be carried out according to the principle of intention to treat all randomised participants as far as is practically possible. If consent to treatment is withdrawn but the participant agrees to remain in the study for follow-up, the participant will be followed until completion. If the participant decides to withdraw consent completely, however, the reasons for withdrawal of consent will be recorded (if possible) and reported for both groups.

The primary outcome is ‘time to failure’. Analysis of time to treatment failure will be summarised by Kaplan-Meier curves for each treatment group and compared overall using the logrank test and survival regression methods. For secondary outcomes, continuous data will be reported as differences in means and binary data will be reported in terms of the relative risk, each with 95% confidence intervals. Missing data will be monitored and strategies developed to minimise their occurrence. Missing data will be handled by considering the robustness of the complete case analysis in relation to sensitivity analysis using various imputation assumptions; however, these analyses will be informed by data collected on the reasons for missing data.

### Economic analysis plan

The cost analysis will be carried out by adopting the perspectives of the National Health Service and personal social service providers and patients, which approximates a societal perspective. Unit cost data will be obtained from appropriate sources [57,58], and total costs for each patient will be calculated.

Two analytic approaches will be used: a within-trial analysis and an economic model. Trial-based estimates of cost-effectiveness will be calculated based on standard methods [59]. Uncertainty in parametric estimates will be addressed by applying nonparametric bootstrapping and estimating cost-effectiveness acceptability curves [59]. We will also apply regression models of cost and outcomes with age, baseline active anterior uveitis grade score and other covariates as deemed appropriate to minimise bias in the estimates of incremental cost-effectiveness.

A model-based extrapolation of the trial results will be performed to explore the impact of a longer analytic time horizon and health outcomes on the treatment cost-effectiveness [60]. The impact of adalimumab on the development of cataracts, glaucoma and blindness will be estimated by constructing risk equations based on epidemiological data [61,62]. A Markov model of treatment effect on JIA will be developed with costs and health state utilities derived from published sources attached to health states to assess the long-term costs and benefits of the two treatment arms.

Incremental cost-effectiveness ratios (ICERs) will be estimated based on quality-adjusted life-year (QALY) estimates. Costs and benefits exceeding 1 year will be discounted at 3.5% per annum in accordance with the current National Institute for Health and Care Excellence rate [63]. Estimates of ICERs will be compared with the £20,000 to £30,000 per QALY threshold for cost-effectiveness [63], and a range of uni- and multivariate analyses, as well as probabilistic sensitivity analyses, will be conducted to assess the robustness of the analysis.

## Trial status

At the time of manuscript submission, this trial was open at 14 hospital sites and had recruited 58 patients.

## Abbreviations

AC: Anterior chamber; ACR: American College of Rheumatology; AE: Adverse event; CMO: Cystoid macular oedema; CRF: Case report form; CTU: Clinical Trials Unit; GCP: Good Clinical Practice; HUI2: Health Utilities Index Mark 2; ICER: incremental cost-effectiveness ratio; ICH: International Conference on Harmonisation; ISRCTN: International Standard Randomised Controlled Trial Number; logMAR: Logarithm of the minimum angle of resolution; MCRN CTU: Medicines for Children Research Network Clinical Trials Unit; OCT: Optical coherence tomography; PI: Principal Investigator; QALY: Quality-adjusted life-year; R&D: Research and development; SAE: serious adverse event; SUN: Standardisation of the Uveitis Nomenclature; TB: Tuberculosis; TNF: Tumour necrosis factor; TNFR: Tumour necrosis factor receptor; ULN: Upper limit of normal.

## Competing interests

The authors declare that they have no competing interests.

## Authors’ contributions

AR and MB are the co-Chief Investigators and have both led all stages of the study design. AD, DB, DD, BH, HH, DH, AJ, PW and CE participated in the writing of the protocol, the design of the case report forms and the preparation of regulatory applications and amendments. SCL has given input into protocol amendments and redesign of case report forms. All of the authors read and approved the final submitted manuscript.

## Authors’ information

AR is a paediatric rheumatologist at Bristol Royal Hospital for Children and Royal National Hospital for Rheumatic Diseases, Bath, UK. AD is Professor of Ophthalmology and Faculty Research Director for Medicine and Dentistry at the Bristol Eye Hospital, Bristol, UK. DD is Research Officer in Health Economics in the Centre for Health Economics & Medicines Evaluation, Bangor University, Bangor, UK. DB is the head of research and innovation at the University Hospitals Bristol NHS Foundation Trust, Bristol, UK. SCL is a consultant rheumatologist at Great Ormond Street Hospital, London. BH is a trial coordinator at the Clinical Trials Research Centre, University of Liverpool, Liverpool, UK. HH is a senior trial manager at the Clinical Trials Research Centre, University of Liverpool, Liverpool, UK. DH is a professor of pharmacoeconomics at the Centre for Health Economics and Medicines Evaluations at Bangor University, Bangor, UK. AJ is a senior statistician at the Clinical Trials Research Centre, University of Liverpool, Liverpool, UK. PW is head of the Centre for Paediatric and Adolescent Rheumatology at University College London and a consultant rheumatologist at Great Ormond Street Hospital, London. CE is a consultant ophthalmologist at Ipswich Hospital, UK, and at Great Ormond Street Hospital, London. MB is Professor of Child Health, University of Liverpool; Academic Lead in the Clinical Academic Department of Paediatric Rheumatology at Alder Hey Children’s NHS Foundation Trust, Liverpool, UK; and Chair of the UK’s NIHR Medicines for Children Research Network/Arthritis Research UK Paediatric Rheumatology Clinical Studies Group.
